# A review of studies on *Oryzias dancena*

**DOI:** 10.1186/s42826-020-00081-x

**Published:** 2021-01-06

**Authors:** In-Seok Park

**Affiliations:** Division of Marine Bioscience, College of Ocean Science and Technology, Korea Maritime & Ocean University, Busan, 49112 Republic of Korea

**Keywords:** Highlight, *Oryzias dancena*, *Review*

## Abstract

*Oryzias dancena* (Beloniformes; Teleostei), is a euryhaline teleost that mainly inhabits the brackish or freshwater of river mouths and estuaries around the Bay of Bengal and the Malay Peninsula. It also has a short interval between generations, with spawning possibilities just 60 days after hatching. The aim of this paper is to provide a review for the study of *O. dancena* based on the studies collected so far, and could form the basis for a wide category of researches including zootoxy, cytogenetics, anesthesia, tagging, salinity tolerance, reproduction, fish disease, chromosome engineering, and trnasgenesis in order to highlight the recent progress in different fields of study using this species.

## Introduction

*Oryzias dancena* (Hamilton, 1822: Asian medaka; Indian ricefish; Tamil-India; Mandaria Chinese-China; marine medaka-Republic of Korea) (*Oryzias dancena* [1, 2] is a freshwater-brackish fish species native to the India, Bangladesh, Sri Lanka, Myanmar and Thailand. Their maximum length is only 3.1 cm (1.2 in.). They are normally found in brackish habitats near the coast, but it lives in fresh water as well [3, 4]. It is not considered threatened^3^.

In various salinity conditions, the development, growth and reproduction of *O. dancena* was higher and better than those of Japanese medaka *O. latipes* [5, 6]. In addition, Inoune and Takei [7] and Kang et al. [8] used the *O. dancena* as a molecular biomarker and an experimental fish for adapting to seawater. The *O. dancena* shows better tolerance than the *O. latipes* including survival rates of adult fish and hatched rates of oosperm in hyperosmotic environments [7, 8]. Generally, mean body length of adult O. dancena is 3–5 cm, and also has a short interval between generations with spawning possible only 60 days after hatching [6, 9, 10].

*Oryzias dancena* isn’t indigenous to Republic of Korea. However, this species is accredited by the Ministry of Land, Transport and Maritime Affairs, Republic of Korea (Ordinance of Agriculture, Food and Fisheries, No. 1 – the law regarding experimental animals, No. 9982) and is imported legally from Indonesia [10]. Recently, the Institute of Marine Living Modified Organisms (*i*MLMOs) selected this species for a living modified organism evaluation project addressing the risks associated with aquatic-LMOs. In line with this purpose, detailed information on the biology of *O. dancena* is beginning to be collected. This review has revealed *O. dancena* from the knowledge based on the studies achieved up to date could form the basis for a wide range of research including zootoxy, cytogenetics, anesthesia, tagging, salinity tolerance, reproduction, fish disease, chromosome engineering, and trnasgenesis using this species (Table 1).

**Table 1:** A review of publications for *Oryzias dancena*

Item	References
**1. Zootaxy**	*Oryzias dancena*-Wikipedia
	Robert (1998) [11]
	Takehana et al. (2005) [12]
	Abraham (2011)
	Froese et al. (2019) [4]
**2. Cytogenetics**	Takehana et al. (2007) [13]
	Takehana et al. (2008) [14]
	Kim et al. (2011) [15]
**3. Anesthesia**	Park et al. (2011) [12]
	Park et al. (2014) [16]
	Park et al. (2017) [6]
**4. Tagging**	Im et al. (2017) [17]
**5. Salinity tolerance**	Inoue and Takei (2003) [7]
	Kang et al. (2008) [8]
	Cho et al. (2010a) [5]
**6. Reproduction**	Song et al. (2009a) [18]
	Song et al. (2009b) [19]
	Cho et al. (2010b) [20]
	Lim et al. (2012) [21]
	Takehana et al. (2014) [22]
	Im et al. (2016) [23]
	Ryu and Gong (2017) [24]
	Park (2020) [25]
**7. Fish disease**	Park et al. (2019) [26]
**8. Chromosome engineering**	Goo et al. (2015) [27]
	Park et al. (2016a) [9]
	Park et al. (2016b) [28]
	Park and Choi (2018) [29]
	Park and Gil (2018) [30]
	Park et al. (2018) [10]
	Park (2019a) [31]
	Park (2019b) [32]
**9. Transgenesis**	Cho et al. (2011) [33]
	Cho et al. (2012) [34]

### Zootaxy

Roberts [11] reported the so-called *Oryzias melastigma* (McClelland, 1839), from India, Bangladesh, Myanmar, and Malaysia by numerous authors beginning with Day (1877), is based mainly or entirely on *Aplocheilus panchax* (Hamilton, 1822). Roberts [11] pointed out India and Bangladesh have two species of *Oryzias*, both large. The deeper-bodied of these two species is reported for the first time as *O. dancena* (Hamilton, 1822). The other is identified as *O. carnaticus* (Jerdon, 1849). Myanmar has one large species, *O. dancena*, and one tiny species, *O. uwai* new species. *Oryzias minutillus* Smith, 1945 and *O. uwai* differ from all other *Oryzias* in having 4/5 instead of 5/6 principal caudal fin rays. *Oryzias uwai* differs from *O. minutillus* in being more conspicuously pigmented and having large, 6-rayed pelvic fins often extending to anal fin origin instead of much smaller and shorter 5-rayed pelvic fins. In Thailand (including its part of the Mekong basin) three species are known: a large estuarine species tentatively identified as *O. javanicus* (Bleeker, 1854) and two tiny inland species, *O. mekongensis* Magtoon & Uwa, 1986, and *O. minutillus. Oryzias minutillus* from many localities are more or less melanoproctic, i.e. have a darkly pigmented genital or vent area not seen in other species. The Mekong basin in Laos has two large species, *O. latipes* (Temminck & Schlegel, 1846) and *O. pectoralis* new species, distinguished by a prominent black blotch on the pectoral fin base, both recently collected in the Nam Theun watershed in central Laos; and two tiny species, *O. mekongensis* and *O. minutillus*. Only one species of *Oryzias* is known from the Mekong delta in Vietnam, the small moderately deep-bodied *O. haugiangensis* new species, with 19–22 anal and 9–10 pectoral fin rays. The Indonesian island of Java has one large species, *O. javanicus* (Bleeker, 1852) with 21–25 anal and 11 pectoral fin rays, and one small species, *O. hubbsi* new species, with only 17–21 anal and 9 pectoral fin rays.

Takehana et al. [12] studied the phylogenetic relationships among medaka fishes of 2 genera, *Oryzias* and *Xenopoecilus*, using the nuclear tyrosinase and mitochondrial 12S and 16S rRNA genes. Of the 23 species currently described for these genera, 13 species of *Oryzias* and 2 species of *Xenopoecilus* were examined. The tree topologies obtained from the nuclear and mitochondrial data were consistent, indicating that *Xenopoecilus* is a polyphyletic genus nested within *Oryzias*. This result suggested the necessity for a systematic study and taxonomic revision of *Xenopoecilus*. The combined data analysis of all data partitions resulted in a well-resolved tree, with most internal branches supported by high statistical values. Based on their combined data phylogeny, Takehana et al. [12] divided the *Oryzias* species into three major species groups, namely the *latipes*, *javanicus*, and *celebensis* groups. These three groups corresponded to the three chromosomal groups (biarmed, monoarmed, and fused chromosome groups) previously proposed from karyological analyses. The phylogeographic pattern suggests historical vicariance between Sulawesi Island and the continental shelf.

### Cytogenetics

Although the sex-determining gene DMY has been identified on the Y chromosome in the medaka (*Oryzias latipes*), this gene is absent in most *Oryzias* species, suggesting that closely related species have different sex-determining genes [13]. Takehana et al. [13] investigated the sex-determination mechanism in *O. dancena*, which does not possess the DMY gene. Since heteromorphic sex chromosomes have not been reported in this species, a progeny test of sex-reversed individuals produced by hormone treatment was performed. Takehana et al. [13] presented sex-reversed males yielded all-female progeny, indicating that *O. dancena* has an XX/XY sex-determination system. To uncover the cryptic sex chromosomes, sex-linked DNA markers were screened using expressed sequence tags (ESTs) established in *O. latipes*. Linkage analysis of isolated sex-linked ESTs showed a conserved synteny between the sex chromosomes in *O. dancena* and an autosome in *O. latipes*. Fluorescence in situ hybridization (FISH) analysis of these markers confirmed that sex chromosomes of these species are not homologous. These findings strongly suggest an independent origin of sex chromosomes in *O. dancena* and *O. latipes.*

Although the sex-determining gene DMY has been identified on the Y chromosome in the medaka, *Oryzias latipes*, this gene is absent in most *Oryzias* species [14]. Recent comparative studies have demonstrated that, in the javanicus species group, *Oryzias dancena* and *Oryzias minutillus* have an XX/XY sex determination system, while *Oryzias hubbsi* has a ZZ/ZW system. Furthermore, sex chromosomes were not homologous in these species [14]. Here, Takehana et al. [14] investigated the sex determination mechanism in *Oryzias javanicus*, another species in the javanicus group. Linkage analysis of isolated sex-linked DNA markers showed that this species has a ZZ/ZW sex determination system. The sex-linkage map showed a conserved synteny to the linkage group 16 of *O. latipes*, suggesting that the sex chromosomes in *O. javanicus* are not homologous to those in any other *Oryzias* species. Fluorescence in-situ hybridization analysis confirmed that the ZW sex chromosomes of *O. javanicus* and *O. hubbsi* are not homologous, and showed that *O. javanicus* has the morphologically heteromorphic sex chromosomes, in which the W chromosome has 4,6-diamino-2-phenylindole-positive heterochromatin at the centromere. Takehana et al. [14] pointed out thier findings suggest the repeated evolution of new sex chromosomes from autosomes in *Oryzias*, probably through the emergence of new sex-determining genes.

Kim et al. [15] cloned the cDNAs that encode the glucocorticoid receptors odGR1 and odGR2 from a euryhaline teleost, the marine medaka, *Oryzias dancena*. The open reading frames of odGR1 and odGR2 encode 790 and 783 amino acids, respectively, and show a sequence identity of 46% with each other. When inter- and intra-species comparisons of the GR domains were made, the N-terminal AF-1 (A/B) and hinge (D) domains showed relatively low identities, whereas the DNA-binding (C) domain (DBD) and ligand-binding (E) domain showed relatively high identities. Through phylogenetic analysis, Kim et al. [15] revealed that odGR1 and odGR2 belong to the teleost GR1 and GR2 groups, respectively. Transfection of odGR1 or odGR2 expression vectors into COS-7 cells along with a reporter vector demonstrated that cortisol and dexamethasone dose-dependently induce transcriptional activity in both GRs. As described in other teleostean fish, the transactivity of odGR2 was more sensitive at far lower concentrations of ligands than the transactivity of odGR1. When treated with aldosterone, the reporter gene was activated in COS-7 cells transfected with odGR2 but not in cells transfected with odGR1. RU486 inhibited transactivation by both GRs, but odGR2 was less sensitive to the inhibitor. Kim et al. [15] found out alterations in coregulators, GRIP-1 and SMILE, mediated transactivation that was more drastic for odGR2 than odGR1. A nine-amino acid insertion (WRARQNTDG) in the DBD of odGR1 had a weak but significant influence on the transactivity of odGR2 with respect to responsiveness to agonists or coregulators. Taken together, these results indicate that the two odGRs possess distinct features not only for ligand sensitivity but also for preferential coregulator recruitment.

### Anesthesia

Park et al. [2] evaluated the anesthetic effects (time required for anesthesia to take effect and recovery time) of two anesthetic agents, clove oil and lidocaine–HCl, on marine medaka, *Oryzias dancena*. Park et al. [2] anesthetized fish at different water temperatures (23 °C, 26 °C and 29 °C) and using different concentrations of clove oil (50 ppm, 75 ppm, 100 ppm, 125 ppm, 150 ppm, and 175 ppm) or lidocaine–HCl (300 ppm, 400 ppm, 500 ppm, 600 ppm, 700 ppm, and 800 ppm). The time required for anesthesia to take effect decreased as both anesthetic concentration and water temperature increased for both clove oil and lidocaine–HCl. To anesthetize marine medaka within approximately 1 min, the optimal concentrations for clove oil were 125 ppm at 23 °C, 100 ppm at 26 °C and 75 ppm at 29 °C and for lidocaine–HCl were 800 ppm at 23 °C and 700 ppm at both 26 °C and 29 °C. Park et al. [2] also compared anesthetic effects in marine medaka of different sizes. Both anesthetic exposure time and recovery time were shorter for smaller fish than for larger fish.

The study of Park et al. [16] demonstrated that heat- and cold-induced anesthesia can be used effectively to anesthetize the marine medaka*, Oryzias dancena*. The cold-anesthesia groups were treated at 4–10 °C and showed anesthesia times of 5.7–23.2 s and recovery times of 56.4–85.4 s with all individuals surviving. The heat-anesthesia groups were treated at 36–42 °C and showed anesthesia times of 6.9–35.2 s and recovery times of 38.4–73.1 s with all individuals surviving. Park et al. [16] reported that when fish were anesthetized by heat and cold, the operculum movement number (OMN) greatly increased because the fish were attempting to overcome unfavorable conditions. The whole-body cortisol level of anesthetized marine medaka returned most closely to that of the control at 6 h after recovery, whereas the whole-body glucose level returned most closely to the control level at 2 h after recovery. Park et al. [16] reported that the results from their study extends previous research and its results should contribute to the safe laboratory handling of marine medaka.

Optimum concentrations of anesthetic clove oil and anesthetic lidocaine-HCl were determined for a species of adult marine medaka, *Oryzias dancena*, over a range of salinity conditions, and investigated in a transport simulation experiment by analyzing various water and physiological parameters [6]. Research of Park et al. [6] indicated that the higher the concentration of anesthetic at each salinity, the shorter the anesthesia time at each salinity. At each concentration, fish were anesthetized slower at water salinities over 10 ppt. Anesthesia time at 10 ppt was faster than any other salinity. In 10 ppt salinity, the dissolved oxygen (DO) concentrations and respiratory frequencies of the clove-oil-administered groups decreased until 48 h, whereas the NH_4_^+^ and CO_2_ concentrations increased until 48 h. In same period, the DO, NH_4_^+^, and CO_2_ concentrations and respiratory frequencies all decreased as the clove oil concentration increased. The trends in the DO, NH_4_^+^, and CO_2_ concentrations and respiratory frequencies in the lidocaine-HCl-administered groups were similar to those in the clove-oil-administered groups.

### Tagging

The study was performed to assess visible implant fluorescent elastomer (VIE) tagging and stress response in marine medaka, *Oryzias dancena* [17]. The experimental fish were anesthetized individually and marked with red, yellow, or green elastomer at each of the following three body locations: (1) the abdomen, (2) the back, and (3) the caudal vasculature. During 12 months, the accumulated survival rates of fish in the experimental treatments were not different among red, yellow, and green elastomers. The experimental fish retained > 85% of the tags injected in the back, > 70% of the tags injected in the caudal vasculature, and > 60% of the tags injected in the abdomen. Im et al. [17] observed that the abdomen site was associated with poor tag retention. For all injected sites, the red and green tags were able to be detected more easily than the yellow tags when observed under both visible and UV lights. Tag readability was lower for the abdomen site than for the other sites (back and caudal vasculature). Thus, Im et al. [17] presented VIE tags were easy to apply to marine medaka (< 1 min per fish) and were readily visible when viewed under UV light.

### Salinity tolerance

Inoue and Takei [7] discussed the utility of Japanese medaka, *Oryzias latipes* and related species for studying mechanisms of seawater (SW) adaptation. In addition to general advantages as an experimental animal such as their daily spawning activity, transparency of embryos, short generation time and established transgenic techniques, Japanese medaka have some adaptability to SW unlike the strictly stenohaline zebrafish, *Danio rerio*. Since other species in the genus *Oryzias* exhibit different degrees of adaptability to SW, comparative studies between Japanese medaka, where molecular-biological and genetic information is abundant, and other *Oryzias* species are expected to present varying approaches to solving the problems of SW adaptation. Inoue and Takei [7] introduced some examples of interspecies comparison for SW adaptabilities both in adult fish and in embryos. *Oryzias* species are good models for evolutionary, ecological and zoogeographical studies and a relationship between SW adaptability and geographic distribution has been suggested by Inoue and Takei [7]. Medaka fishes may thus deliver new insights into our understanding of how fish have expanded their distribution to a wide variety of osmotic environments.

To provide more evidence of the hypothesis, two medaka species, *Oryzias latipes* and *O. dancena*, whose primary natural habitats are fresh water (FW) and brackish water (BW) environments, respectively, were compared from levels of mRNA to cells in this study [8]. The plasma osmolalities of *O. latipes* and *O. dancena* were lowest in the FW individuals. The muscle water contents of *O. latipes* decreased with elevated external salinities, but were constant among FW-, BW-, and seawater (SW)-acclimated *O. dancena*. Expression of NKA, the primary driving force of ion transporters in gill ionocytes, revealed different patterns in the two *Oryzias* species. The highest NKA α-subunit mRNA abundances were found in the gills of the SW *O. latipes* and the FW *O. dancena*, respectively. The pattern of NKA activity and α-subunit protein abundance in the gills of *O. latipes* revealed that the FW group was the lowest, while the pattern in *O. dancena* revealed that the BW group was the lowest. Immunohistochemical staining showed similar profiles of NKA immunoreactive (NKIR) cell activities (NKIR cell number × cell size) in the gills of these two species among FW, BW, and SW groups. Taken together, *O. latipes* exhibited better hyposmoregulatory ability, while *O. dancena* exhibited better hyperosmoregulatory ability. Kang et al. [8] results corresponding to the hypothesis indicated that the lowest branchial NKA activities of these two medaka species were found in the environments with salinities similar to their natural habitats.

Cho et al. [5] examined osmoregulatory capabilities of a euryhaline medaka, *Oryzias dancena* (Beloniformes; Teleostei), with a particular emphasis on adult and larval viability during direct salinity changes. *O. dancena* adults were highly capable for hyper-osmoregulation as well as hypo-osmoregulation, as evidenced by no adverse effect on their viability during the direct transfer either from complete freshwater (0‰) to 40‰ salinity, or from 70 to 0‰. Furthermore, the phased increase of external salinity with acclimation periods allowed them to survive at a salinity as high as 75‰. However, tolerant capability to acute salinity increase in early larval stage was much less than in adult stage, based on the finding that the tolerance range of salinity increase was only 15‰ from freshwater, indicating that the hyper-osmoregulation system might not be fully developed in the early larval stage. On the contrary, the hypoosmoregulation system could be more solidified in *O. dancena* larvae, as evidenced by their good survival even after direct transfer from 45 to 0‰.

### Reproduction

Song et al. [18] investigated sex differentiation and gonad development in a marine medaka species, *Oryzias dancena* (Beloniformes; Teleostei). The average time to hatch was 11 days post-fertilization (dpf) at 25 °C. Primordial germ cell (PGC) was first observed at 5 dpf and migrated to presumptive gonadal area between the gut and pronephric duct at 9 dpf. Male and female gonads were morphologically differentiated at 12 days post-hatching (dph). Early oocytes at perinucleolus stage as well as the formation of spermatid and efferent duct were observed at 28 dph. At 6 weeks of age, the ovary exhibited yolk granulation in many oocytes, while testis possessed a considerable number of spermatogonia and spermatids. The first ovulation was observed in 9-week-old females, and at the same age, males contained fully-matured spermatozoa. From the data obtained in their study, Song et al. [18] indicated that the gonad differentiation of *O. dancena* is the typical type of differentiated gonochorism.

Song et al. [19] observed the egg development and morphological changes of larvae, juveniles and adults of *Oryzias dancena*. Fertilized eggs were incubated at 25 ± 1 °C; the process of embryonic development was observed by light microscopy and based on diagnostic features of the developing embryos. The average time to hatch was 11 days after fertilization. The hatched larvae averaged 4.40 ± 0.24 mm in total length (TL). The yolk sacs of the larvae were almost absorbed at 3 days after hatching and 4.55 ± 0.23 mm TL. At 21 days after hatching, the larvae were 7.23 ± 0.73 mm TL and had reached the juvenile stage. First ovulation was about 9 weeks after hatching and at 22.58 ± 2.73 mm TL.

Cho et al. [20] examined the effects of different salinity levels on spawning performance, embryonic development and early viability of a euryhaline medaka species, *Oryzias dancena*. *O. dancena* were able to spawn eggs in a wide range of salinity from 0 to 70‰, however, the spawning frequency was lowered in complete freshwater (0‰) and in highly salted water (70‰). Fertilization success was negatively affected when the environmental salinity was higher than the salt concentration found in normal seawater. Embryonic viability and hatching success were also inversely related with the salinity levels [20]. Typical abnormality was observed in developing embryos incubated at high salinities (30, 45 and 60‰). In addition, the time to hatch was significantly delayed with increasing salinities: peak hatching occurred at 12 ~ 14 days post fertilization (dpf) in freshwater and at least at 17 to 18 dpf in 60‰. Mean survival rates of the hatched larvae up to 7 days post hatching (dph) were at least 97% in salinity levels ranging from 0 to 30‰. However, larvae reared in 45 and 60‰ experienced significant mortality, especially in the early phase, resulting in only 75 and 64% survival rates up to 7 dph, respectively.

Lim et al. [21] observed the reproductive behavior of the marine medaka, *Oryzias dancena,* and determined the factors of reproductive behavior to provide useful information for improving their artificial reproduction techniques. The reproductive behavior of the marine medaka was observed in laboratory aquaria. Once the experiment began, all of the males chased the females. The males attempted to stimulate the urogenital openings of the females. While chasing a female, a large male would bite a relatively small male’s anus. Larger males expelled smaller males with biting, and the defeated males were barred from the female. After the other males were expelled, the remaining male approached and drew alongside the female. The male’s dorsal and anal fins covered the female’s body. Spawning began after complete covering took place. Spawning of males and females occurred simultaneously. The loadings for 2 factors were calculated. The calculation was restricted to 2 factors because these 2 factors explained about 81% of the total common variance and the following factors possessed no practical significance. Two movements (biting, expelling) had high positive values for factor one. This factor related a male’s defensive behavior to courtship behavior and spawning, and explained 23.1% of the total common variance. The second factor had high positive values for chasing, rejection, covering, and parallel swimming. This factor related a male’s courtship behavior and female’s defensive behavior to spawning, and explained 59.7% of the total common variance.

Sex chromosomes harbour a primary sex-determining signal that triggers sexual development of the organism [22]. However, diverse sex chromosome systems have been evolved in vertebrates [22]. Takehana et al. [22] used positional cloning to identify the sex-determining locus of a medaka-related fish, *Oryzias dancena*, and found that locus on the Y chromosome contains a cis-regulatory element that upregulates nighbouring *Sox3* expression in developing gonad. Sex-reversed phenotypes in *Sox3*^*Y*^ transgenic fish, and *Sox3*^*Y*^loss-of-function mutants all point to its critical role in sex determination. Furthermore, Takehana et al. [22] demonstrated that *Sox3* initiates testicular differentiation by upregulating expression of downstream *Gsdf*, which is highly conserved in fish sex differentiation pathways. The results of Takehana et al. [22] not only provided strong evidence for the independent recruitment of *Sox3* to male determination in distantly related vertebrates, but also provided direct evidence that a novel sex determination pathway has evolved through co-option of a transcriptional regulator potentially interacted with a conserved downstream component.

Im et al. [23] examined possible sexual dimorphism and the relative growth patterns of morphometric characteristics in the marine medaka, *Oryzias dancena* for their potential to help differentiate between males and females of this species. The von Bertalanffy growth parameters estimated by a non-linear regression method were L_∞_ = 30.2 mm, K = 3.22/year, and τ_0_ = − 0.05. All 18 characteristics measured showed a difference between males and females from 70 days after hatching. Each of these characteristics were different between sexes, and the ratio of standard length between sexes showed that males were larger than females for all five morphometric measurements. Fin length measurements were taken for 21 distances of anal fin and 7 distances of dorsal fin between landmarks. There were all differences for all dorsal fin rays between the males and the females and there is difference in 70 days after their hatch when the sexual dimorphism is presented. The difference in fin ray for male and female was more greatly seen as they grow. Male marine medaka showed more rapid growth than females, with longer length, dorsal fins and anal fins.

Optimizing the conditions for stem cell culture is an essential prerequisite for the efficient utilization of stem cells [24]. In the culture of fish embryonic stem cells (ESCs) or ESC-like cells, embryo extracts are important for stable growth, but there is no rule for determining the developmental stage of the embryos used to obtain extracts [24]. Therefore, Ryu and Gong [24] investigated the effects of the developmental stage of extract donor embryos on the culture of *Oryzias dancena* ESC-like cells. *O. dancena* ESC-like cells were cultured in different media containing each of four types of embryo extract depending on the developmental stage of the extract donor embryos. Growth, morphology, colony-forming ability, alkaline phosphatase (AP) activity, and embryoid body (EB) formation of the cells were investigated. While the developmental stage of the extract donor embryos did not influence the growth, morphology, AP activity, or EB formation of ESC-like cells, colony-forming ability was affected and the pattern of the effects differed completely between the two ESC-like cells investigated. These results suggest that the developmental stage of extract donor embryos should be selected carefully for the culture of ESC-like cells, according to the research purpose and type of cell line [24].

The method of natural spawning is very passive and inconvenient for the study of developmental engineering in marine medaka, *Oryzias dancena* [25]. The optimum concentration of human chorionic gonadotropin (HCG) and carp pituitary extract (CPE) for ovulation and spawning, and the injection time for the artificial spawning of marine medaka were analyzed by Park [25]. The success rate, survival rate, and hatching rate were highest with 100 IU HCG kg-1 BW and 5 mg CPE L-1 in both male and female marine medaka (*p* < 0.05). After obtaining unfertilized eggs and sperm by the injection of HCG and CPE into the broodstock of marine medaka, artificial fertilization could be successfully achieved any time fertilized eggs are needed in this species. This result by Park [25] should be useful for developing a study program for marine medaka as an experimental animal.

### Fish disease

Park et al. [26] investigated the sterilization effects of methylene blue (MB), formalin, and iodine on the egg of marine medaka, *Oryzias dancena*, for disinfecting naididae worm, *Chaetogaster diastrophus* through sterilization. To determine harmfulness of MB, formalin, and iodine, lethal concentrations 50 (LC_50_) of three chemicals were analyzed in the eggs of marine medaka. The sterilized periods of each chemical were set at 1 h. Sterilized rates of naididae worm in each chemical were affected and increased drastically as the concentration of each chemical increased. Sterilization abilities of naididae worm were most effective for formalin, but survival rates of egg and hatched rates for formalin were lowest among each chemical. The LC_50_ of MB over 96 h were 185.26, 103.84, and 127.15 ppm for adults, juveniles, and eggs respectively. The toxic effects of MB were clearly dose dependent for each life stage. The toxicity sensitivity of juveniles to MB was dramatically higher than that of other groups [24]. In 48 h after sterilization, cortisol and glucose concentrations of the adult group with MB treatment were higher than those of the adult group with no treatment.

### Chromosome engineering

Goo et al. [27] determined the influence of triploidization on cell and nucleus size characteristics of the same tissues of erythrocyte, retina, kidney, hepatocyte and midgut epithelium in marine medaka, *Oryzias dancena* histologically. Induced triploid fish are produced by cold shock treatments. Likewise, the size of horizontal cell nucleus in inner nuclear layer of retina, ganglion cell nucleus in ganglion cell layer of retina, proximal tubule cell of kidney, hepatocytes and nuclear height of midgut epithelium all appear to be larger than diploid. On the other hand, retina thickness is larger in diploid than induced triploid. Induced triploid shows low density of cell number. Results of their study Goo et al. [27] suggested that same characteristics in the induced triploid exhibiting larger cells and nucleus sizes with fewer number of cells than the diploid can be useful criteria for the distinction between diploid and induced triploid, and also the ploidy level in marine medaka.

Park et al. [9] induced triploidy in the marine medaka, *Oryzias dancena* by cold shock treatment (0 °C) of fertilized eggs for 45 min, applied two minutes after fertilization. The diploid and triploid male fish were larger than their female counterparts, and the concentrations of thyroid stimulating hormone (TSH) and thyroxine (T_4_) were higher in the induced triploids over 1 year. In both the diploid and triploid groups the concentrations of TSH and T_4_ were higher in the male fish than in the females, while the testo-sterone and estradiol-17ß concentrations in the induced triploids were lower than in the diploids. The gonadosomatic index (GSI) of the triploid fish was lower than that for the diploids, and the GSI for females in each ploidy group were higher than that for the males. For both groups the GSI was highest at 4 months of age, and decreased thereafter to 12 months. Analysis of the gonads of one-year-old triploid fish suggested that the induction of triploidy probably causes sterility in this species; this effect was more apparent in females than in males.

Park et al. [28] induced triploidy in the marine medaka, *Oryzias dancena* by cold shock treatment (0 °C) of fertilized eggs for 30, 45 or 60 min, applied two minutes after fertilization. The triploid genotype was induced by each of the thermal shock regimes tested. The best result was obtained when the eggs were treated for 45 min, which induced triploidy in all the resulting fish. Triploidy was confirmed using chromosomal and flow cytometer analyses, and erythrocyte measurements. The surface areas and volumes of the erythrocytes of triploid fish were significantly larger than those of diploid fish, and their chromosome number (3 *N* = 72) was 1.5 times greater that for the diploids (2 *N* = 48). Based on a flow cytometer analysis, the triploid fish had approximately 1.5 times the cellular DNA content (2.40 pg/ cell) of the diploid specimens (1.61 pg/cell).

Park and Choi [29] observed the differences of erythrocyte nucleus between diploid and induced triploid in Far Eastern catfish, *Silurus asotus* and marine medaka, *Oryzias dancena*. The types of atypical cell were determined three types in induced triploid Far Eastern catfish; asymmetric division, irregular-shaped and absence of nucleus. The types of atypical cell were determined three types in induced triploid marine medaka; snippety nucleus, sickle-shaped and absence of nucleus. In Far Eastern catfish and marine medaka, the numbers of atypical cells in induced triploid were higher than those of diploid. The absence of nucleus in diploid and induced triploid were shown in Far Eastern catfish and marine medaka, and this type in diploid samples was the lowest among the other types in both species. Occurrence frequency of nucleus’s absence and total number of atypical cells in induced triploid Far Eastern catfish was higher than that in induced triploid marine medaka.

Park and Gil [30] examined the fluctuating asymmetry of eye diameter, maxilla length, operculum length, and the number of pectoral fin ray and pelvic fin ray between ploidy and sex in diploid and triploid marine medaka, *Oryzias dancena*. In all experimental groups, eye diameter and maxilla length showed no difference between left side and right side. Results of operculum length in triploid male group and pectoral fin ray’s number in diploid male group showed similarity ones with results of operculum length in triploid female group and pectoral fin ray’s number in diploid female group. However, the operculum length in diploid male group and pectoral fin ray’s number in triploid male group showed considerable difference with those of operculum length in diploid female group and pectoral fin in triploid female group. Findings of pelvic fin ray’s number in all groups were similar to those of pectoral fin ray’s number in all groups.

Park et al. [10] examined the morphometric truss characteristics and classical dimensions of the marine medaka, *Oryzias dancena*, that might distinguish diploid and triploid fish. Differences in all the classical and truss dimensions of the diploid and triploid fish were observed in both sexes. All the dimensions of the triploid fish were greater than those of the diploid fish. The triploid marine medaka shows sexual dimorphism in these characters, and the sexual dimorphism of the triploid marine medaka is similar to that of the diploid marine medaka. Thus, when their classical dimension and truss dimension was measured, the growth of triploid marine medaka is faster than that of the diploid fish, and it displays clear sexual dimorphism, with male fish having longer dorsal and anal fins than female fish.

The sectioned-body morphometric characteristics of the diploid and triploid marine medaka, *Oryzias dancena*, of both sexes were examined by Park [31] to collect basic data on the significant differences between the diploid and triploid fish. Significant differences between the diploid and triploid fish in both sexes were observed by Park [31] in the body circumference anterior to the base of the pelvic fin, the body circumference anterior to the base of the anal fin, the body circumference anterior to the base of the dorsal fin, the area anterior to the base of the pelvic fin, the area anterior to the base of the anal fin, the area anterior to the base of the dorsal fin, the total height anterior to the base of the pelvic fin, the total height anterior to the base of the anal fin, the height anterior to the base of the pelvic fin, the height anterior to the base of the anal fin, the width anterior to the base of the anal fin, the belly thickness II anterior to the base of the anal fin, section shape 2–1, and section shape 4–1 (*p* < 0.05). Park [31] reported these measurements were greater in the triploid marine medaka of both sexes than those in the diploid marine medaka of both sexes, and they were also greater in the male diploid and triploid marine medaka than those in the corresponding female fish. Park [31] concluded that the sectioned-body morphometric dimensions were greater in the triploid males than those in the triploid females and the diploid fish in this study.

Eggs of the marine medaka, *Oryzias dancena* were collected and fertilized to observe the temperature–salinity-related cleavage rates and mitotic intervals (**τ**_0_) [32]. Park [32] investigated the relationship between **τ**_0_ and five different water temperatures (18, 22, 26, 30, and 34 °C) and four different salinities (0, 10, 20, and 30 ppt NaCl). As the water temperature increased, the slope of the first cleavage frequency increased with elapsed time after fertilization, and approximately 30% of fertilized eggs reached the first cleavage frequency at every 15-min intervals. However, the slope of the first cleavage frequency did not differ between 0 ppt and the other salinities (10, 20, or 30 ppt). At higher water temperatures, the eggs developed more rapidly, but no other developmental process was affected. At higher salinity, the hatching rates of the eggs decreased and the hatching times were delayed. There was a strong negative correlation between **τ**_0_ and water temperature as shown in this eq. (Y = − 1.137X + 75.47, R2 = 0.977, where Y is **τ**_0_ and X is water temperature). At a constant water temperature, **τ**_0_ did not differ in 0, 10, 20, and 30 ppt NaCl.

### Transgenesis

The cytoskeletal β-actin gene was characterized from marine medaka, *Oryzias dancena* and the functional capability of its promoter to drive constitutive expression of foreign reporter protein was evaluated [33]. The *O. dancena* β-actin gene possessed a conserved genomic organization of vertebrate major cytoplasmic actin genes and the bioinformatic analysis of its 5′-upstream regulatory region predicted various transcription factor binding motifs. Heterologous expression assay using a red fluorescent protein (RFP) reporter construct driven by the *O. dancena* β-actin regulator resulted in stunningly bright expression of red fluorescence signals in not only microinjected embryos but also grown-up transgenic adults. Although founder transgenics exhibited mosaic patterns of RFP expression, transgenic offspring in subsequent generations displayed a vivid and uniform expression of RFP continually from embryos to adults. Based on the blot hybridization assays, two transgenic lines established in this study were proven to possess high copy numbers of transgene integrants (approximately 240 and 34 copies, respectively), and the transgenic genotype in both lines could successfully be passed stably up to three generations, although the rate of transgene transmission in one of the two transgenic lines was lower than expected Mendelian ratio. Significant red fluorescence color could be ubiquitously observable in all the tissues or organs of the transgenics. Quantitative real-time RT-PCR represented that the expression pattern of transgene under the regulation of β-actin promoter would resemble, in overall, the regulation of endogenous β-actin gene in adult tissues, although putative mechanism for competitive or independent regulation between transgene and endogenous gene could also be found in several tissues. Results from Cho et al. [33] study undoubtedly indicated that the *O. dancena* β-actin promoter would be powerful enough to fluorescently visualize most cell types in vivo throughout its whole lifespan. Cho et al. [33] reported that their study could be a useful start point for a variety of transgenic experiments with this species concerning the constitutive expression of living fluorescent color reporters and other foreign proteins.

Cho et al. [35] established the stable transgenic germlines carrying the red fluorescence protein (RFP) gene (rfp) driven by fast skeletal myosin light chain-2 gene (mlc2f) promoter in a truly euryhaline fish species, the marine medaka (*Oryzias dancena*; Beloniformes). Transgenic lines contained transgene copy numbers varying from a single copy to more than 230 copies per genome. Although the transgenic founders displayed mosaic and/or ectopic expression of the RFP signal, the resultant F1 transgenics and their progeny showed consistently stable transmission of the transgenic locus and uniform RFP signal through several subsequent generations. In adult transgenics, an authentic brilliant red fluorescence was achieved over the skeletal muscles of the transgenic individuals, which might be sufficient for ornamental display. Expression analysis of the transgenic mRNAs indicated that rfp transcripts were predominantly expressed in the skeletal muscles. Different transgenic lines displayed different levels of transgene expression at the mRNA, protein, and phenotypic levels. However, the efficiency of transgene expression was independent of the transgene copy number. The RFP protein levels were consistently stable in the transgenic fish muscles through several generations, up to F5. Cho et al. [35] pointed out the results of their study suggest that transgenic marine medaka that acquire strong fluorescent signals in their skeletal muscles can be developed as a promising, novel ornamental fish for display in both freshwater and seawater aquaria.

## Conclusions

*Oryzias dancena* inhabits brackish water or freshwater around The Bay of Bengal and Malay peninsula, and this species is not indigenous to Republic of Korea. This fish is a truly euryhaline teleost with a great capacity for hypo- and hyper-osmoregulation. It also has a short interval between generations with spawning possibilities just 60 days after hatching. This review has covered a variety of studies for *O. dancena* based on the studies collected so far, and could form the basis for a wide category of researches including zootoxy, cytogenetics, anesthesia, tagging, salinity tolerance, reproduction, fish disease, chromosome engineering, and trnasgenesis in order to highlight the recent progress in different fields of study using this species.

## Data Availability

Available.
